# Editorial: Maternal-fetal interface: new insight in placenta research

**DOI:** 10.3389/fendo.2023.1325568

**Published:** 2023-11-28

**Authors:** Cilia Abad, Mariana Farina, Alicia E. Damiano, Reinaldo Marín

**Affiliations:** ^1^ Department of Pharmacology and Toxicology, Faculty of Pharmacy in Hradec Kralove, Charles University, Hradec Králové, Czechia; ^2^ Faculty of Medicine, Center of Pharmacological and Botanical Studies (CEFYBO-CONICET), University of Buenos Aires, Buenos Aires, Argentina; ^3^ Department of Biological Science, Faculty of Pharmacy and Biochemistry, University of Buenos Aires, Buenos Aires, Argentina; ^4^ Institute of Physiology and Biophysics Bernardo Houssay 018(IFIBIO Houssay), CONICET, University of Buenos Aires, Buenos Aires, Argentina; ^5^ Center for Biophysics and Biochemistry (CBB), Venezuelan Institute for Scientific Research (IVIC), Caracas, Venezuela

**Keywords:** pregnancy, placenta, maternal-fetal health, maternal-fetal interface, placental pathology

Pregnancy, a complex and multifaceted biological process, often goes underappreciated despite involving intricate mechanisms to ensure the well-being of both the mother and the fetus. The placenta, often regarded as the bridge between mother and baby, plays a central role in this process. Recent studies have shed light on the importance of placental health, revealing potential therapeutic targets (1).

The objective of the Research Topic “*Maternal-Fetal Interface: New Insights in Placenta Research*” was to bring together original research articles and reviews that highlight recent advances in understanding the functions of the placenta and its impact on the health of both the mother and her future child. This Research Topic includes a diverse range of studies, encompassing ten original articles, six reviews, and one systematic review.

One significant concern is obesity, affecting approximately 30% of expectant mothers in the US (2). Its adverse effects on pregnancy, including issues like mitochondrial dysfunction and placental inflammation, may be linked to reduced Vitamin D levels. Encouragingly, Vitamin D supplementation emerges as a potential remedy, offering a relatively straightforward solution to a complex problem.

Furthermore, maternal hypothyroidism is associated with fetal growth, placental dysfunction, and reduced kisspeptin/Kiss1R at the maternal-fetal interface (3, 4). In this regard, daily treatment with kisspeptin improves fetal development and placental morphology in an experimental model of hypothyroid rats, blocking placental oxidative damage, and increasing the expression of growth factors and antioxidant enzymes in the placenta.

Chronic histiocytic intervillositis (CHI) is a rare placental lesion associated with recurrent pregnancy issues (5). A systematic review and meta-analysis aimed to understand the perinatal consequences of CHI pregnancies and the potential benefits of treatment. While various drugs show potential, more research is needed to validate their safety, efficacy, and optimal dosage.

Shiga toxin-producing *Escherichia coli* (STEC) in the endocervix of asymptomatic pregnant women could have a potential role in adverse pregnancy outcomes (6). Bacterial findings reveal that a significant percentage of asymptomatic pregnant women with STEC in their endocervical samples. These findings suggest that STEC may be present in the lower female reproductive tract during pregnancy, raising new questions about its potential impact on pregnancy complications.

The impact of COVID-19 on pregnant women has been a cause for concern (7, 8). Findings suggest that certain molecules are upregulated in placentas exposed to COVID-19, indicating a potential innate defense mechanism of the placenta against the SARS-CoV-2 virus.

Advanced techniques have allowed for the study of microRNAs (miRNAs) in placenta accreta, revealing altered expressions and potential regulatory networks (9). Similarly, research into the serotonin system in the human placenta has illuminated its potential pathways and alterations, which could have consequences for the fetus.

Oxysterols, small molecules with a potentially monumental role in maternal-fetal health, hypertension during pregnancy, and the placental serotonin system are other areas being thoroughly investigated (10). The research aims to detect oxysterols and their subsequent metabolites in the placenta, umbilical cord blood plasma, maternal plasma, and amniotic fluid. This study contributes to a deeper understanding of the role of these molecules at the maternal-fetal interface.

Placental dysfunction can lead to gestational hypertension (11). In fact, it is well established that defective placental development/function is the root cause of early-onset preeclampsia and fetal growth restriction (12). Protein modifications (O-GlcNAcylation) are linked to hypertension, but their impact on placental function remains unclear (13). Female Wistar and spontaneously hypertensive rats (SHR) were studied during pregnancy, revealing that SHR had higher blood pressure, smaller fetuses, and reduced placental efficiency. Morphological changes in the placenta were observed, with lower O-GlcNAc protein expression and enzyme levels in hypertensive rats. This suggests that insufficient placental O-GlcNAcylation impairs fetal growth by disrupting placental function.

Environmental factors, especially air pollution, have been shown to pose risks to pregnancy, emphasizing the need for global efforts to improve air quality (14).

The pattern of glycan expression at the maternal-fetal interface could have a crucial role in driving the physiological alterations during pregnancy (15). Glycans’ role emerges as a key player in understanding disorders like preeclampsia. On the other hand, lipids and fatty acids are essential elements in the metabolic processes within the human placenta, actively participating in fetal development (16). Dysregulated lipid metabolism and abnormal functioning of lipases in the placenta have been associated with various pregnancy-related complications, including conditions like preeclampsia and preterm birth. In this regard, the significance of diacylglycerol lipase β (DAGLβ) highlights the importance of intracellular lipases in lipid network regulation, with potential implications for placental function in normal and compromised pregnancies.

Addressing complications like fetal intrauterine growth restriction (IUGR), which results from impaired trophoblast syncytialization, requires a deeper understanding of the involved regulators (17). Machine learning is innovatively employed to identify patterns and predict perinatal disorders, marking a new era in maternal and fetal healthcare.

The successful implantation of a blastocyst in a healthy pregnancy relies on the decidualization of uterine endometrial stromal fibroblast cells (hESF) (18). MicroRNAs (miRs) play a critical role in cellular function and can influence recipient cells. Investigating how decidualization affects miR release by hESF, with a focus on miR-19b-3p linked to recurrent pregnancy loss, reveals that *in vitro* decidualization reduces the release of several miRs. Notably, miR-19b-3p is elevated in the endometrium of patients with a history of early pregnancy loss. Functionally, miR-19b-3p overexpression impedes trophoblast cell proliferation and increases HOXA9 expression, suggesting that miR release by decidualized hESF may regulate other cell types in the decidua, emphasizing the importance of proper miR release for healthy implantation and placental development.

Nutrient transport, especially in conditions like gestational diabetes mellitus (GDM), plays a crucial role (19). Leptin’s role in modulating this transport, particularly in GDM, emerges as a critical research avenue.

Traditional Chinese medicine (TCM) offers potential solutions for complications like GDM (20). The discovery of the significance of ferroptosis in GDM pathogenesis and the potential therapeutic properties of *Coptis chinensis* underscore the harmonious integration of traditional knowledge and modern science.

In conclusion, placental health is a diverse and intricate field. Advances in research, a deeper understanding of molecular mechanisms, and innovative solutions are converging to shape a brighter future for maternal and fetal health. Interdisciplinary collaboration and a commitment to scientific excellence hold the promise of better interventions and therapies. We aspire to a future where every pregnancy is celebrated with the expectation of a healthy and thriving outcome, free from complications and disease.

This journey of discovery and understanding opens the door to innovations that can transform maternal-fetal health, offering hope and happiness for all mothers and unborn children. It is a future that honors the miracle of life in all its wondrous and complex forms, where each step brings us closer to realizing a vision of a brighter and healthier future for all.

## Author contributions

CA: Writing – original draft, Writing – review & editing. MF: Writing – original draft, Writing – review & editing. AD: Writing – original draft, Writing – review & editing. RM: Writing – original draft, Writing – review & editing.
